# Spontaneous Spinal Epidural Hematoma Complicated by Pulmonary Embolism During Pregnancy: A Case Report

**DOI:** 10.3389/fmed.2022.832693

**Published:** 2022-03-25

**Authors:** Wei Jiang, Xuan-Yu Tan, Jia-Ai Li, Ming Dong

**Affiliations:** ^1^Department of Neurology and Neuroscience Center, The First Hospital of Jilin University, Changchun, China; ^2^Department of Neurosurgery, The First Hospital of Jilin University, Changchun, China

**Keywords:** spontaneous spinal epidural hematoma, pulmonary artery embolism, methylprednisolone therapy, conservative treatment, pregnancy

## Abstract

**Background:**

Spontaneous spinal epidural hematoma, without discernable underlying conditions, is considered a neurological emergency, and is rare during pregnancy.

**Case Presentation:**

We report the case of a 24-year-old patient at 37 weeks of gestation. She had back pain that progressed to paraplegia of both lower limbs within 2 days. Thoracic magnetic resonance imaging revealed a lesion behind the spinal cord at the T5–T6 level, suggestive of spontaneous spinal epidural hematoma. Due to the rapid recovery of muscle strength in her lower limbs after an emergency cesarean section, we used methylprednisolone therapy to reduce spinal edema rather than decompression of the spinal canal. We incidentally found that the patient’s left pulmonary artery was occluded. In consideration of spontaneous spinal epidural hematoma as relative contraindication to anticoagulation, and in the absence of pulmonary embolism symptoms, including good partial oxygen pressure, we did not administer anticoagulant therapy. The patient’s condition improved rapidly in the following week.

**Conclusion:**

Spontaneous spinal epidural hematoma concomitant with pulmonary artery embolism is an extremely rare manifestation during pregnancy. As exemplified by our case, desirable treatment outcomes are possible under such cases.

## Introduction

Spontaneous spinal epidural hematoma (SSEH) is a rare condition, defined as a hematoma that occurs in the spinal epidural space without any precipitating factors (1). The usual clinical presentation includes sudden neck or back pain that progresses to paraparesis or quadriparesis, depending on the site of the lesion (2). Standard treatment consists of urgent operative decompression, and conservative management may be advisable for patients who show spontaneous recovery within a short period (3, 4). Pregnancy is a hypercoagulable stasis and vascular damage state, which increases the risk of pulmonary embolism (PE) (5). Societal guidelines state that computed tomography pulmonary angiography (CTPA) positivity requires anticoagulant therapy in patients (6). However, when pulmonary embolism was diagnosed in our patient, she had severe symptomatic SSEH. In the light of mild pulmonary embolism symptoms and good blood oxygen partial pressure, after weighing the advantages and disadvantages, we decided not to administer anticoagulant therapy. The patient’s satisfactory outcome following methylprednisolone (MP) therapy instead of surgical decompression suggests an alternative treatment method for SSEH. To the best of our knowledge, this is the first case of SSEH complicated by a pulmonary embolism during pregnancy.

## Case Description

**FIGURE 1:**
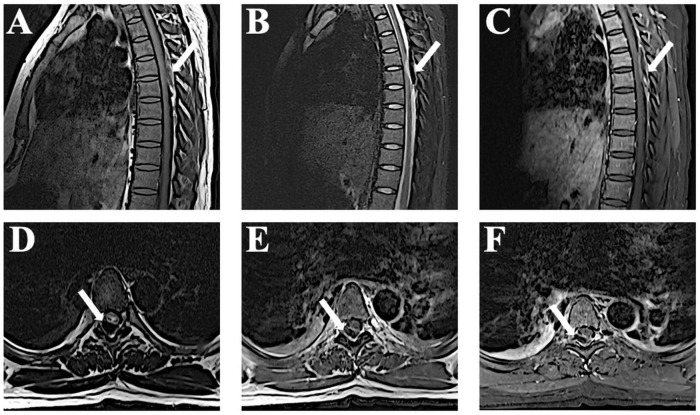
Magnetic resonance imaging (MRI) observations at admission. **(A,B)** Sagittal T1-weighted magnetic resonance images reveal a lesion at T5–T6 that appears as an isointense signal and T2-weighted shows a hypointense signal. **(C)** The edge of the hematoma shows mild enhancement after gadolinium administration. **(D–F)** Axial images show the lesions accordingly.

A 24-year-old patient at 37 weeks’ gestation presented with a 2-day history of back pain; she developed paraplegia of both lower limbs and urinary incontinence 1 day later. The patient was in good health and had no history of underlying diseases including coagulopathy. Physical examination after admission showed absence of lower extremity reflexes, no proprioception, and complete sensory loss bilaterally to the T6 level. The Medical Research Council (MRC) scale evaluation indicated a lower limb strength score of 0/5. We performed an urgent thoracic magnetic resonance imaging (MRI) that showed SSEH at the T5–T6 level (Figure 1). On T1-weighted images, the lesion was hyperintense and heterogeneous, while on T2-weighted images, the signals were hypointense and heterogeneous in the spinal cord.

**FIGURE 2:**
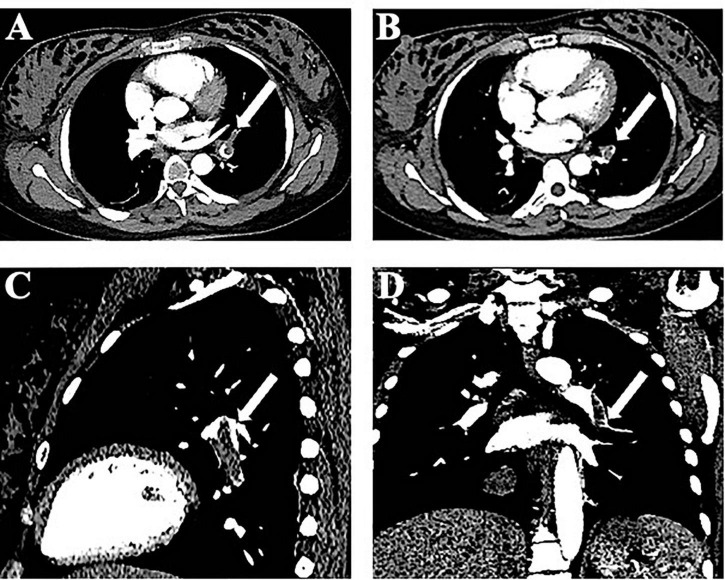
Imaging of computed tomography pulmonary angiography. **(A)** Computed tomography pulmonary angiography (CTPA) shows an embolism at the left pulmonary artery (axial images). **(B)** CTPA shows right pulmonary artery branch embolism. **(C,D)** Sagittal and coronal images.

As the patient was 37 weeks pregnant, the obstetrician suggested the performance of a cesarean section before spinal canal decompression. Six hours after the cesarean section, the patient began to recover muscle strength in the lower limbs, and the MRC scale evaluation indicated a lower limb strength score of 2/5. Due to the rapid recovery of muscle strength, we chose not to perform spinal decompression. The patient underwent spinal artery computed tomography angiography (CTA) for examination of blood vessels of the spinal cord, and we were informed that the patient had PE. An emergency CTPA was performed, and left pulmonary artery trunk and small pulmonary artery emboli in each lobe of both lungs were identified (Figure 2). D-dimer levels had increased to 3,567 μg/L. Color ultrasonography of the lower extremity showed no abnormalities. We also conducted autoimmune laboratory tests and reviewed the relevant coagulation routine test results, which showed no significant abnormalities. Unusually, the patient exhibited no symptoms of pulmonary embolism, such as dyspnea. Blood gas analysis revealed a partial oxygen pressure of 96 mmHg. Further test results, including blood oxygen saturation, were also normal.

**FIGURE 3:**
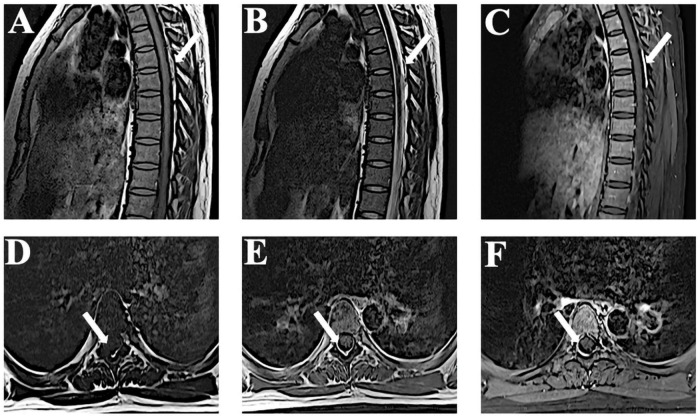
Time-dependent MRI observations. **(A–F)** Follow-up MRI performed 9 days after admission reveals that the lesion is significantly reduced in T1-weighted, T2-weighted, and enhanced images. MRI, magnetic resonance imaging.

**FIGURE 4:**
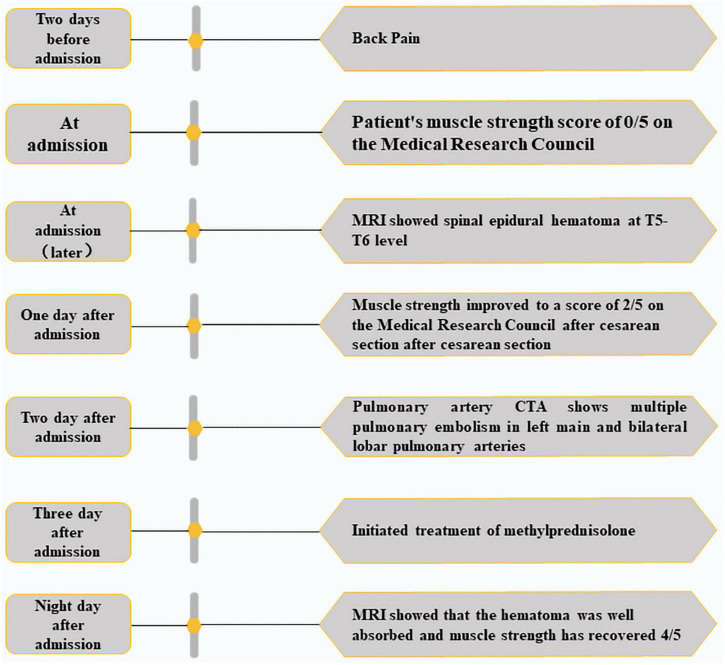
Timeline of historical and current information from this case.

We administrated intravenous methylprednisolone (dose, 10 mg/kg/day for the 5 days) with no adverse reactions. On day 9 after admission, the MRI showed that the hematoma had been absorbed (Figure 3). The patient’s lower limb muscle strength recovered to 4/5. The follow-up at 2 years showed that neurological function had completely recovered. A CTA of the pulmonary artery was also normal. A summary of the timeline is presented in Figure 4.

## Discussion

Potential causes of SSEH include coagulopathies, increased venous pressure, anticoagulant therapy, vascular malformation, and pregnancy (7). Pregnancy-induced hemodynamic changes, as well as structural changes in the vascular walls, may be an important part of the pathogenesis of SSEH (8). The study of Groen et al. supports the theory that SSEH is attributable to the rupture of a spinal epidural vein in the venous plexus encircling the spinal dura rather than an arterial abnormality (9). In addition, Nathoo et al. (10) showed that the vertebral venous plexus is a fragile structure. It is valveless, and intrathoracic and intraspinal pressure mounts when intra-abdominal pressure increases (10). During the late stages of pregnancy, the venous abdominal/pelvic dynamics are altered. This fluctuation in venous pressure may lead to a rupture of pre-existing venous walls (11). SSEH is most often located in the thoracic spine area, because the epidural venous plexus mainly protrudes in the thoracic spine (12), which is consistent with the focus of our case. However, the rapid progression of SSEH symptoms cannot be excluded as being of arterial origin. The source of arterial bleeding in a cervical epidural hematoma is the free anastomotic artery that runs in the epidural space and connects with the nerve root artery (13).

Rapid decompression of the spinal canal is considered the standard treatment for most SSEHs. It may be beneficial to perform the spinal cord decompression surgery after the cesarean section because early delivery of the fetus reduces epidural venous congestion, which is conducive to decompression (14). However, non-operative treatments have been reported to result in good outcomes in patients with mild symptoms or those with rapid recovery (15). This may be explained by spontaneous decompression of the hematoma within the epidural space (4). Measures such as MP administration may result in prompt improvements (16). MP is a neuroprotective drug used to treat spinal cord injury and compression, and can reduce spinal edema through anti-inflammatory properties. As in our case, the patient in a previous study (16) reported a significant improvement in muscle strength within 6 h after the cesarean section, and conservative treatment was considered reasonable. However, conservative treatment is only recommended for patients who recover within a short period of time. The conservative treatment plan should include close observation in the neurosurgery ward and timely repetition of an MRI. Surgical intervention may still be necessary if symptoms return and resolution of the hematoma ceases.

Acute PE is considered as one of the leading causes of maternal death, and its significant risk factors include venous thromboembolism, obesity, medical comorbidities, stillbirth, pre-eclampsia, postpartum hemorrhage, and cesarean section (17). The patient had embolism not only in the main pulmonary artery, but also in some small branches, hence it is difficult to explain the PE on the basis of venous thrombosis alone. Therefore, we speculate that this may be related to a variety of factors, such as hypercoagulability after cesarean section and SSEH leading to restricted movement of lower limbs after paraplegia. Anticoagulant therapy is considered to be a risk factor for SSEH. A study stated that patients develop SSEH after undergoing anticoagulant therapy (18); therefore, we believe that the use of anticoagulants may aggravate the development of SSEH. Moreover, SSEH was described as major bleeding (19), which is a contraindication to anticoagulation; consequently, it seems reasonable that we did not use low molecular weight heparin even after main pulmonary artery occlusion. Most importantly, the patient showed no obvious symptoms, such as dyspnea, which provided us the choice to monitor the patient’s neurological and respiratory symptoms, instead using anticoagulant therapy.

The case of conservative treatment of SSEH associated with oral anticoagulant therapy in a child supports the premise that patients who present with SSEH without focal neurological deficits can be successfully managed while maintaining therapeutic levels of anticoagulants (20). In this case, close follow-up with frequent neurological examinations, imaging, and monitoring of the prothrombin time is mandatory. In SSEH treatment, the selection of the treatment scheme by doctors with respect to administration of anticoagulation should be based on the specific condition of the patients.

The lack of subsequent dynamic evolution of MRI was a limitation of this study. The patient underwent repeat CTPA and spinal cord MRI at the local hospital; however, we were unable to access it.

In conclusion, we report a rare case of pregnancy complicated by SSEH and pulmonary embolism, with satisfactory outcome of the patient following MP treatment instead of surgical decompression. When there is a disagreement with respect to SSEH and pulmonary embolism treatment, anticoagulant therapy can be appropriately delayed or omitted depending upon the clinical condition of the patient.

## Data Availability Statement

The original contributions presented in the study are included in the article/supplementary material, further inquiries can be directed to the corresponding author.

## Ethics Statement

The patients/participants provided their written informed consent to participate in this study. Written informed consent was available from the individual(s) for the publication of any potentially identifiable images or data included in this article.

## Author Contributions

WJ, X-YT, and J-AL contributed to the design of this case report, recorded the medical information, and drafted the manuscript. MD assessed the data and made revisions to the manuscript. All authors contributed to the critical revision and provided final approval for the submitted version of this manuscript.

## Conflict of Interest

The authors declare that the research was conducted in the absence of any commercial or financial relationships that could be construed as a potential conflict of interest.

## Publisher’s Note

All claims expressed in this article are solely those of the authors and do not necessarily represent those of their affiliated organizations, or those of the publisher, the editors and the reviewers. Any product that may be evaluated in this article, or claim that may be made by its manufacturer, is not guaranteed or endorsed by the publisher.
